# Identification of anthocyanin biosynthesis genes in rice pericarp using PCAMP

**DOI:** 10.1111/pbi.13133

**Published:** 2019-05-15

**Authors:** Xinghai Yang, Xiuzhong Xia, Zongqiong Zhang, Baoxuan Nong, Yu Zeng, Yanyan Wu, Faqian Xiong, Yuexiong Zhang, Haifu Liang, Yinghua Pan, Gaoxing Dai, Guofu Deng, Danting Li

**Affiliations:** ^1^ Rice Research Institute Guangxi Academy of Agricultural Sciences Nanning China; ^2^ Biotechnology Research Institute Guangxi Academy of Agricultural Sciences Nanning China; ^3^ Cash Crops Research Institute Guangxi Academy of Agricultural Sciences Nanning China

**Keywords:** multiple pools, whole‐genome resequencing, SNP‐index, anthocyanin, candidate genes

Anthocyanins are a kind of biologically active flavonoids, which have strong anti‐oxidation and anti‐mutation functions as phytonutrients and have important effects on human health. The anthocyanin metabolic pathway has been extensively studied in *Arabidopsis thaliana*,* Petunia hybrida* and *Zea mays*, which involves many structural genes and regulatory genes. However, only a few anthocyanin biosynthesis‐related genes have been identified in rice, such as *Rd* (Furukawa *et al*., 2007), *OsCHI* (Hong *et al*., 2012) *and Kala4* (Oikawa *et al*., 2015). The traditional method of mapping quantitative trait loci (QTLs) is only for two corresponding alleles and is time‐consuming and labour‐intensive. High‐throughput sequencing technologies have become the new strategies for mapping the important traits of crops, such as simultaneous mapping and mutation identification by deep sequencing (SHOREmap) (Schneeberger *et al*., 2009), next‐generation mapping (NGM) (Austin *et al*., 2011), mutation mapping (MutMap) (Abe *et al*., 2012), QTL‐seq (Takagi *et al*., 2013) and genome‐wide association study (GWAS) (Liu and Yan, 2019) can rapidly identify the genes for plant traits. However, SHOREmap requires a much larger sample size; the NGM studies the genes belongs to the recessive homozygous mutant phenotype; MutMap mainly identifies the single gene‐controlled quality traits; QTL‐seq constructs only two pools showing extreme opposite trait values for a given phenotype in a segregating progeny and maps 1–2 major genes for target trait; GWAS is applicable to natural population with a large sample size and thus its cost is high, and it is also difficult to detect the rare mutations and minor effective genes.

Here, we introduced Pair‐wise Comparison Analysis for Multiple Pool‐seq (PCAMP), an optimized method of QTL‐seq to identify the genomic candidate regions involved in anthocyanin biosynthesis in rice pericarp. In this protocol, the second filial generation (F_2_) progeny generated by crossing two parents with different target traits were divided into n (n ≥ 3) subpopulations according to their phenotypes. Thirty phenotypically identical individuals were selected from each subpopulation, and their DNA samples were extracted to form a pool for sequencing. Finally, we compared the SNP‐index between every two Pool‐seq to map the genomic candidate regions.

**Figure 1:**
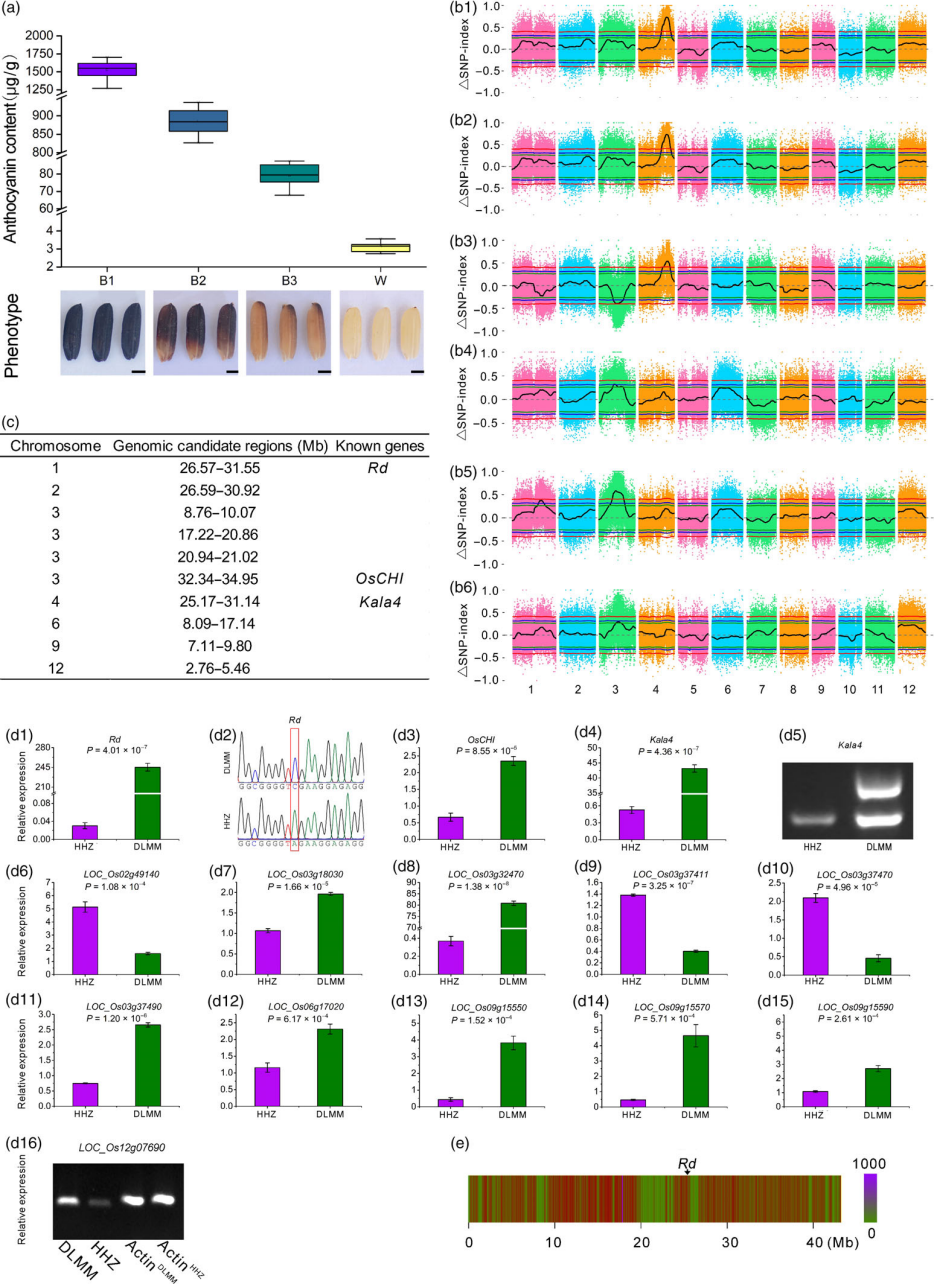
(a) The phenotype and anthocyanin content in four pools of F^2^ population. Four pools: B1, B2, B3 and W. Scale bars, 1.2 mm. (b) The PCAMP approach for mapping genomic regions controlling anthocyanin biosynthesis genes in rice pericarp. (b1) ▵SNP‐index plots between W and B1, (b2) W and B2, (b3) W and B3, (b4) B1 and B2, (b5) B1 and B3, and (b6) B2 and B3. The *y*‐axis is the name of the chromosome, coloured dots represent the calculated SNP‐index, and the black line is the fitted SNP‐index. The green line, blue line and red line represent the threshold of 90%, 95% and 99% confidence interval, respectively. (c) The final genomic candidate regions of anthocyanin biosynthesis in rice pericarp. (d) The candidate genes of anthocyanin biosynthesis in rice pericarp. (d1, d3, d4, d6–d15) The expression levels of 13 genes through qPCR. (d2) DNA sequencing of *Rd* and the 43rd base of the second exon is mutated from C to A. (d5) Amplification of *Kala4* using functional primers. (d16) The expression levels of *LOC_Os12g07690* through RT‐PCR. (e) The *Rd* is located within the low‐density SNP regions on chromosome 1.

Donglanmomi (DLMM) is a rice variety with high anthocyanin content (1797.82 μg/g DW). It was crossed to Huanghuazhan (HHZ) with very low anthocyanin content (3.68 μg/g DW) to generate F_1_ progeny, and F_2_ progeny were derived from self‐pollination of the F_1_ progeny. After the rice seeds were fully matured, the progeny segregated in a 601:195 ratio for coloured pericarp and white pericarp phenotypes, respectively, conforming to a 3:1 segregation ratio (chi‐squared test: χ^2^ = 0.11, nonsignificant) and indicating that a gene plays an important role in anthocyanin biosynthesis in rice pericarp. Previous research showed that this gene was *Kala4* (Oikawa *et al*., 2015). Subsequently, the F_2_ progeny were divided into four subpopulations according to the anthocyanin content of 796 individuals, and the DNA samples of 30 individuals in each subpopulation were mixed in equal amounts to form four pools: B1, B2, B3 and W, respectively (Figure 1a).

The DNA of DLMM, HHZ, B1, B2, B3 and W was sequenced using Illumina HiSeq X Ten high‐throughput sequencing technology. After data filtration, the total base of six samples together was 161.48 Gb; of which, DLMM, HHZ, B1, B2, B3 and W accounted for 36.55 Gb, 39.63 Gb, 22.11 Gb, 21.64 Gb, 21.89 Gb and 19.66 Gb, respectively. Single nucleotide polymorphisms (SNPs) of six samples were detected through GATK software.

To identify the genomic candidate regions responsible for anthocyanin biosynthesis in rice pericarp, we compared the SNP‐index between any two different pools. Distance method was used to fit the ΔSNP‐index, and the distribution of ΔSNP‐index is shown in Figure 1b1–b6. For the genomic candidate regions with overlapping physical positions on the same chromosome, the intersection regions were selected as the final genomic candidate regions. Therefore, the regions showing a significant association with anthocyanin biosynthesis‐related genes in rice pericarp are shown in Figure 1c.

Three genomic candidate regions were adjacent to or contained the cloned genes of anthocyanin biosynthesis (Figure 1c). *Rd* was found to be involved in the proanthocyanidin biosynthesis of rice pericarp (Furukawa *et al*., 2007). The expression levels of *Rd* between DLMM and HHZ were significantly different (Figure 1d1). The sequences of DLMM and HHZ were amplified with PCR primer (F: ccatcaccaagtgcaaggta, R: agtcgtcgtggtcgtaggag), and the products were sequenced. The 43rd base of the second exon of the *Rd* of HHZ was changed from C to A causing premature termination of translation of mRNA (Figure 1d2). Why is *Rd* located at the upstream of the genomic candidate region (1.19 Mb)? The number of SNPs in the genomic region nearby *Rd* was greatly reduced (Figure 1e). Thus, the false‐positive result may be resulted from a decrease in nucleotide polymorphism within this genomic region.

*OsCHI* is a key gene involved in flavonoid metabolic pathway (Hong *et al*., 2012). The expression levels of *OsCHI* between DLMM and HHZ were significantly different (Figure 1d3).

*Ra* is located in the candidate region on chromosome 4, which encodes the basic helix–loop–helix (bHLH) transcription factor, which plays a regulatory role in the anthocyanin biosynthesis (Hu *et al*., 1996). Subsequently, Hu *et al*. (2000) indicated that *Ra* consisted of *Ra1* and *Ra2*. Recently, Oikawa *et al*. (2015) successfully cloned *Kala4*, a key gene responsible for anthocyanin accumulation in rice pericarp, which was found to be the same gene as *Ra2*. The expression levels of *Kala4* between DLMM and HHZ were significantly different (Figure 1d4). The DNA of DLMM and HHZ was amplified by functional primers (F: agggagtctctgtccggttacgtc, R1: cggtgttagggccccatctatcc, R2: gccgttcgtcaatc acaagcgtc). The results showed that the promoter region of *Kala4* in DLMM had a genomic fragment inserted (Figure 1d5), and this change was the causes of generation of the black rice traits (Oikawa *et al*., 2015).

There are 61 SNPs with ΔSNP‐index ≥ 0.67 in 26.59–30.92 Mb on chromosome 2. They included a homozygous variant site of ΔSNP‐index = 1. The expression levels of *LOC_Os02g49140* between DLMM and HHZ were significantly different (Figure 1d6), and this gene encodes glycosyltransferase. In the anthocyanin biosynthetic pathway, glycosylation modification affects its stability in cells.

Within the 8.76‐ to 10.07‐Mb region on chromosome 3, there are 24 SNPs with ΔSNP‐index ≥0.67 and two homozygous variant loci with ΔSNP‐index = 1. The expression levels of *LOC_Os03g18030* between DLMM and HHZ were significantly different (Figure 1d7). This gene encodes leucoanthocyanidin dioxygenase, a key enzyme involved in anthocyanin biosynthetic pathway in plants.

In the region of 17.22–21.02 Mb on chromosome 3, there were 4620 SNPs with ΔSNP‐index ≥0.67, including 69 homozygous variant sites with ΔSNP‐index = 1. The expression levels of *LOC_Os03g32470*,* LOC_Os03g37411*,* LOC_Os03g37470* and *LOC_Os03g37490* (Figure 1d8–d11) between DLMM and HHZ were significantly different. *LOC_Os03g32470* encodes leucoanthocyanidin dioxygenase, which catalyses the oxidative dehydration of leucocyanidins to form the anthocyanins. The other three genes encode MATE efflux family protein. *LOC_Os03g37411* and *LOC_Os03g37490* are highly homologous to *AtTT12* of *Arabidopsis thaliana*. In *Arabidopsis thaliana*,* AtTT12* is involved in the transport of anthocyanins or proanthocyanidins to vacuoles. In addition, *TT12* also plays an important role in the flavonoid metabolism pathways in rape and cotton.

There were 96 SNPs with ΔSNP‐index ≥0.67 in 8.09–17.14 Mb on chromosome 6, including two homozygous mutation sites of ΔSNP‐index = 1. The expression levels of *LOC_Os06g17020* between DLMM and HHZ were significantly different (Figure 1d12). *LOC_Os06g17020* encodes anthocyanin 3‐O‐beta‐glucosyltransferase, a key enzyme catalysing the oxidation of unstable anthocyanidins into anthocyanins.

There were seven SNPs with ΔSNP‐index ≥0.67 in the candidate region on chromosome 9, and the expression levels of *LOC_Os09g15550*,* LOC_Os09g15570* and *LOC_Os09g15590* (Figure 1d13–d15) between DLMM and HHZ were significantly different. These three genes all encode F‐box domain‐containing protein.

There were 40 SNPs with ΔSNP‐index ≥0.67 in 2.76–5.46 Mb on chromosome 12, including a homozygous variation site of ΔSNP‐index = 1. The expression levels of *LOC_Os12g07690* between DLMM and HHZ were significantly different (Figure 1d16). The function of *LOC_Os12g07690* is related to flavonoid biosynthesis.

In this study, we applied PCAMP to F_2_ populations and successfully identified 10 genomic candidate regions involved in anthocyanin biosynthesis in rice pericarp; among them, the genes *Rd*,* OsCH*, and *Kala4* have been cloned. The results showed that the PCAMP method may be a powerful tool for identifying multiple gene‐controlled traits in rice.
